# Comparative Molecular Transporter Properties of Cyclic Peptides Containing Tryptophan and Arginine Residues Formed through Disulfide Cyclization

**DOI:** 10.3390/molecules25112581

**Published:** 2020-06-02

**Authors:** Eman H. M. Mohammed, Dindyal Mandal, Saghar Mozaffari, Magdy Abdel-Hamied Zahran, Amany Mostafa Osman, Rakesh Kumar Tiwari, Keykavous Parang

**Affiliations:** 1 Center for Targeted Drug Delivery, Department of Biomedical and Pharmaceutical Sciences, Chapman University School of Pharmacy, Harry and Diane Rinker Health Science Campus, Irvine, CA 92618, USA; emohammed@chapman.edu (E.H.M.M.); mandal@chapman.edu (D.M.); mozaf100@mail.chapman.edu (S.M.); 2 Chemistry Department, Faculty of Science, Chemistry department, Menoufia University, Shebin El-Koam 51132, Egypt; magdyzahran@gmail.com (M.A.-H.Z.); amanyosman812@gmail.com (A.M.O.); 3 Department of Chemistry, College of Science and Humanities in Al-Kharj, Prince Sattam Bin Abdulaziz University, Al-Kharj 11942, Saudi Arabia

**Keywords:** cell-penetrating peptide, cancer, cytotoxicity, cellular uptake, disulfide bridge, drug delivery, phosphopeptide

## Abstract

We have previously reported cyclic cell-penetrating peptides [WR]_5_ and [WR]_4_ as molecular transporters. To optimize further the utility of our developed peptides for targeted therapy in cancer cells using the redox condition, we designed a new generation of peptides and evaluated their cytotoxicity as well as uptake behavior against different cancer cell lines. Thus, cyclic [C(WR)_x_C] and linear counterparts (C(WR)_x_C), where x = 4–5, were synthesized using Fmoc/*t*Bu solid-phase peptide synthesis, purified, and characterized. The compounds did not show any significant cytotoxicity (at 25 µM) against ovarian (SK-OV-3), leukemia (CCRF-CEM), gastric adenocarcinoma (CRL-1739), breast carcinoma (MDA-MB-231), and normal kidney (LLCPK) cells after 24 and 72 h incubation. Both cyclic [C(WR)_5_C] and linear (C(WR)_5_C) demonstrated comparable molecular transporter properties versus [WR]_5_ in the delivery of a phosphopeptide (F′-GpYEEI) in CCRF-CEM cells. The uptake of F′-GpYEEI in the presence of 1,4-dithiothreitol (DTT) as the reducing agent was significantly improved in case of l(C(WR)_5_C), while it was not changed by [C(WR)_5_C]. Fluorescence microscopy also demonstrated a significant uptake of F′-GpYEEI in the presence of l(C(WR)_5_C). Cyclic [C(WR)_5_C] improved the uptake of the fluorescent-labeled anti-HIV drugs F′-d4T, F′-3TC, and F′-FTC by 3.0–4.9-fold. These data indicate that both [C(WR)_5_C] and linear (C(WR)_5_C) peptides can act as molecular transporters.

## 1. Introduction

Efficient molecule translocation across the cell membrane remains a major obstacle for intracellular cargo delivery. The cell membrane is made of mucopolysaccharides and phospholipids that are negatively charged and very hydrophobic. The nature of the cell membrane makes it very impermeable for many water-insoluble or negatively-charged molecules. For example, phosphopeptides are used as probes for studying phosphoprotein–protein interactions; the major challenge is to transport this negatively charged molecule into the cells [1,2,3]. Furthermore, a variety of anticancer and anti-HIV agents have limited therapeutic applications due to poor cellular uptake. Translocation of cell impermeable compounds, such as phosphopeptides, into cells requires vectors that facilitate cell binding and internalization. The development of highly effective cellular delivery systems to enhance the delivery of biologically important cargos has become an urgent need. Numerous delivery systems have been explored, such as liposomes and micelles. While important progress in cargo delivery has been made with these various vectors, none of them are optimal [4,5].

Recently, the use of cell-penetrating peptides (CPPs) in drug delivery has dramatically increased in basic research and preclinical studies [6,7,8]. CPPs have been shown to facilitate the passage of different cargos across the cell membrane, enhance the cellular uptake of the drugs and afford improved bioactivity. CPPs are efficient cellular uptake vectors, due to their low toxicity, physiological stability, and efficiency for rapid delivery into cells [9,10]. Depending on their physicochemical properties, CPPs can be cationic, amphipathic, or hydrophobic [11]. Amphipathic CPPs’ sequences contain an alternating pattern of polar and non-polar regions. Poly-arginine peptides display the highest level of cellular uptake due to the presence of the guanidine unit that has a tendency to form bidentate hydrogen bonding with the negatively-charged moieties, such as the carboxylic, sulfate, and phosphate groups of cell membrane proteins in mucopolysaccharides and phospholipids, respectively, leading to cellular internalization of peptides under physiological conditions [12]. Besides arginine, the presence of other amino acids has shown an efficient enhancement of cellular uptake. The addition of four tryptophan residues to a CPP’s sequence has shown to improve its cellular internalization [13].

Positively charged CPPs bind to the cell surface via electrostatic interaction with proteoglycans called glycosaminoglycans (GAG), which are negatively charged and presented everywhere in the cell membranes and form a platform that connects CPPs or the CPP/cargo complex to the extracellular matrix [14,15,16]. The secondary structure, number, location of arginine in the CPP’s sequence, and the chirality of amino acids are the main factors associated with enhanced uptake efficiency [17,18,19,20,21].

Cyclic CPPs are a unique emerging class of conformationally constrained peptides with a higher metabolic stability and binding specificity to their targets. The cyclization of linear peptides reduces the degree of freedom, and this constrained structure provides specificity for their target and higher binding affinity. These cyclic CPPs permeate the membrane through a number of different mechanisms, and some may require multiple pathways [22]. The properties of amino acids in cyclic CPPs provide unique membrane permeability and transporter properties [23]. Prior studies showed that cyclic amphipathic peptides composed of alternative tryptophan (W) and arginine (R) residues, [WR]_4_ and [WR]_5_, enhanced the cellular uptake of some cell-impermeable compounds, such as anti-HIV drugs, doxorubicin, phosphopeptides, and siRNA, or generated peptide nanostructures [23,24,25,26,27,28] by us and others [22]. Cyclic peptides have high stability towards proteolytic degradation and more efficiency for cargo delivery due to the rigidity of cyclic peptides [28].

In continuation of our efforts to develop a structure–cellular uptake relationship for cyclic peptides composed of tryptophan and arginine residues, herein we designed a modification to our previously developed peptide with disulfide cyclization as a new generation of peptides and evaluated their cytotoxicity and cellular uptake against different cancer cell lines. The rationale of current studies was that cancer cells have a higher percentage of glutathione as compared with normal cells [29,30]. Thus, disulfide bonds can be reduced in cancer cells significantly higher than normal cells, providing the selective delivery of anticancer drugs to cancer cells.

## 2. Results and Discussion

### 2.1. Chemistry

**Figure 1:**
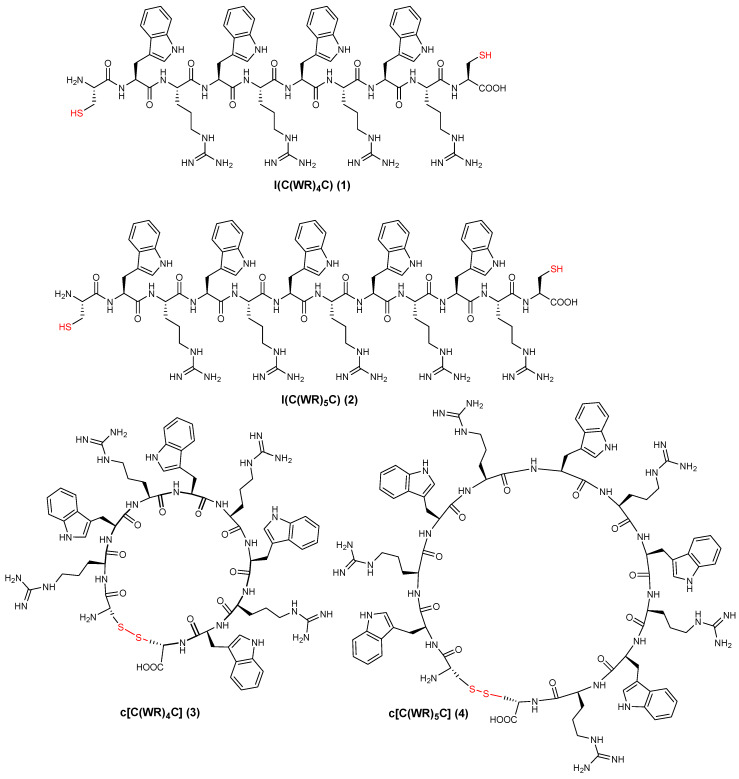
Chemical structures of synthesized peptides.

**Scheme 1:**
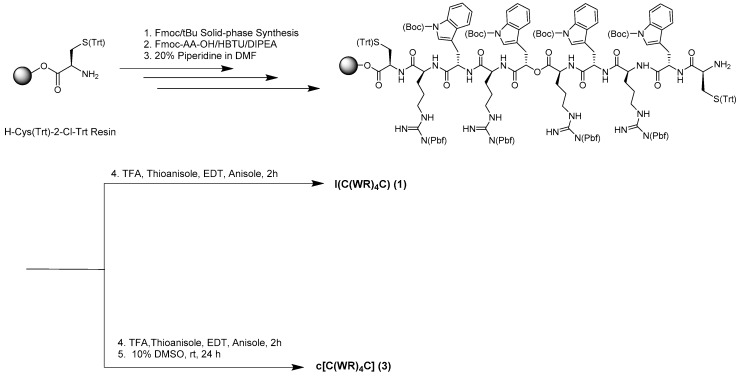
Synthesis of l(C(WR)_4_C) (**1**) and c[C(WR)_4_C] (**3**) as representative examples.

Four linear and cyclic peptides were synthesized using Fmoc/*t*Bu solid-phase peptide synthesis (Figure 1). These series of peptides were designed to determine the impact of the changes in the cyclization strategy on the cellular uptake and cytotoxicity. The synthetic procedure for the synthesis of l(C(WR)_4_C) and c[C(WR)_4_C] is shown in Scheme 1 as representative examples. Parentheses () and brackets [] represent the linear and cyclic peptides, respectively. Further, letters l and c represent linear and cyclic peptides, respectively. The linear peptide containing alternative tryptophan (W) and arginine (R) amino acids with two cysteines (C) residues (CWRWRWRWRC) was assembled on the cysteine preloaded chlorotrityl resin using coupling and deprotecting reagents as depicted in Scheme 1. The linear peptide was completely cleaved in the presence of a freshly prepared cleavage cocktail containing trifluoroacetic acid (TFA)/thioanisole/1,2-ethanedithiol (EDT)/anisole (90:5:3:2, *v*/*v*/*v*/*v*) to afford l(C(WR)_4_C). The linear peptide was subjected to an oxidation reaction using a 10% DMSO-H_2_O solution over 24 h at room temperature to afford a disulfide-linked cyclic peptide. Peptides were characterized and purified using matrix-assisted laser desorption/ionization (MALDI) mass spectroscopy and reverse-phase high-performance liquid chromatography (RP-HPLC), respectively. The analytical HPLC data (in Supplementary Materials) showed a retention time (RT) of 9.6 and 9.8 min for the linear peptides (C(WR)_4_C) and (C(WR)_5_C), respectively, while the RTs for cyclic peptides [C(WR)_4_C] and [C(WR)_5_C] were 10.3 and 10.5 min, respectively.

### 2.2. In Vitro Cytotoxicity Assay of Peptides

**Figure 2:**
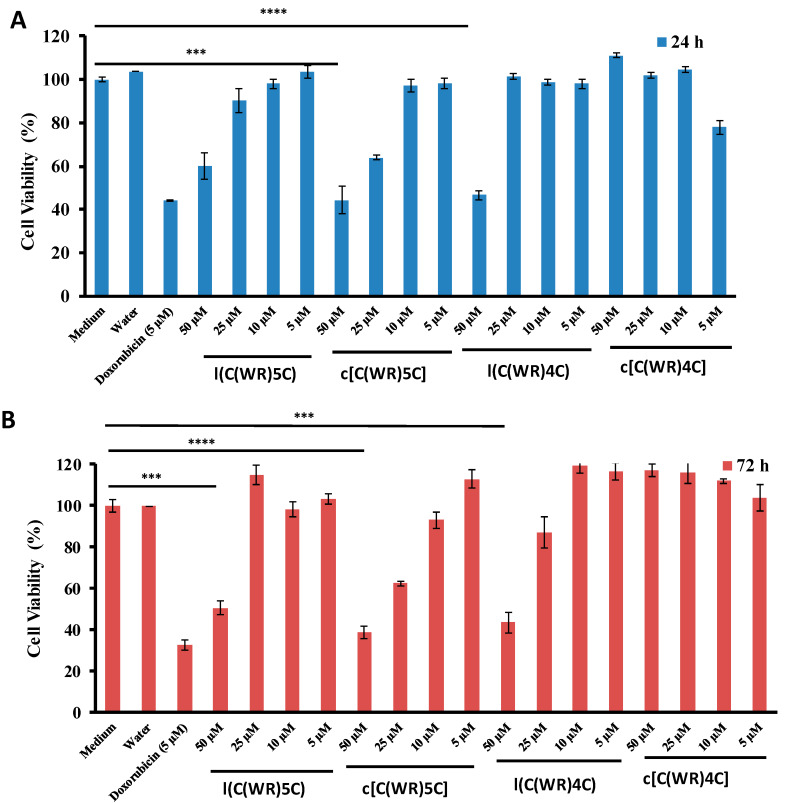
MTS assay of peptides (5–50 µM) on LLC-PK1 cell lines. (**A**) 24 h incubation and (**B**) 72 h incubation (*n* = 3) (if *p* < 0.05 then *, if *p* < 0.01 then **, if *p* < 0.001 then ***, if *p* < 0.0001 then ****). Error bars are SD.

**Figure 3:**
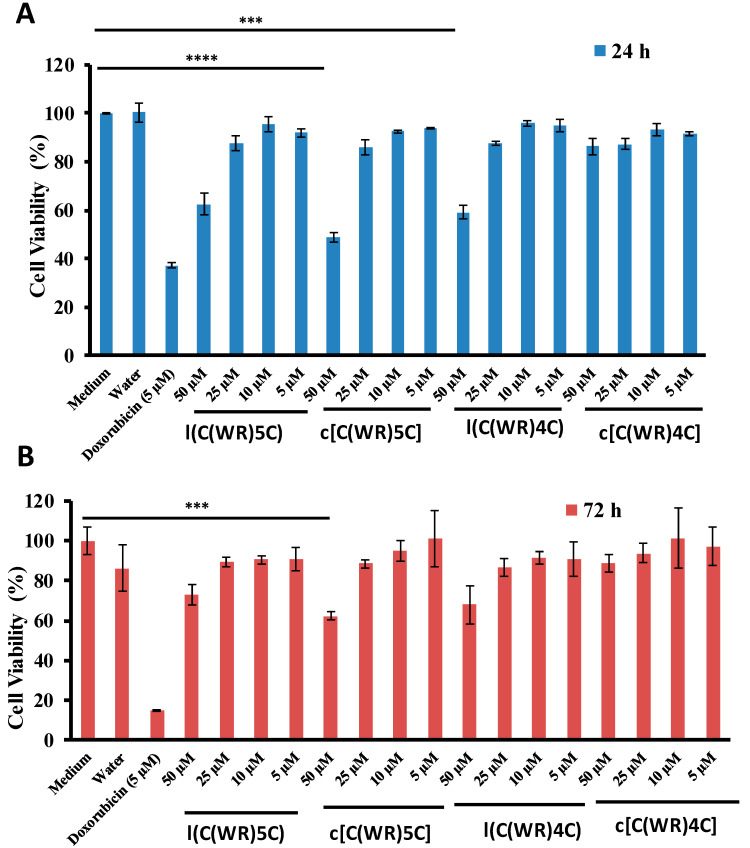
MTS assay of peptides (5–50 µM) on CCRF-CEM cell lines: (**A**) 24 h incubation and (**B**) 72 h incubation (*n* = 3) (if *p* < 0.05 then *, if *p* < 0.01 then **, if *p* < 0.001 then ***, if *p* < 0.0001 then ****). Error bars are SD.

**Figure 4:**
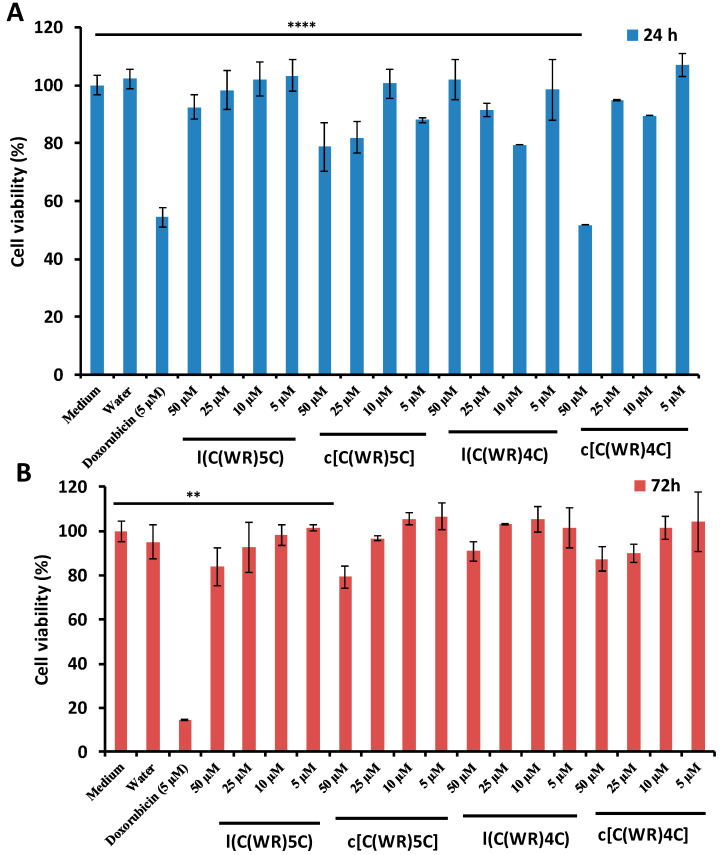
MTS assay of peptides (5–50 µM) on CRL-1739 cell lines: (**A**) 24 h incubation and (**B**) 72 h incubation (*n* = 3) (if *p* < 0.05 then *, if *p* < 0.01 then **, if *p* < 0.001 then ***, if *p* < 0.0001 then ****). Error bars are SD.

**Figure 5:**
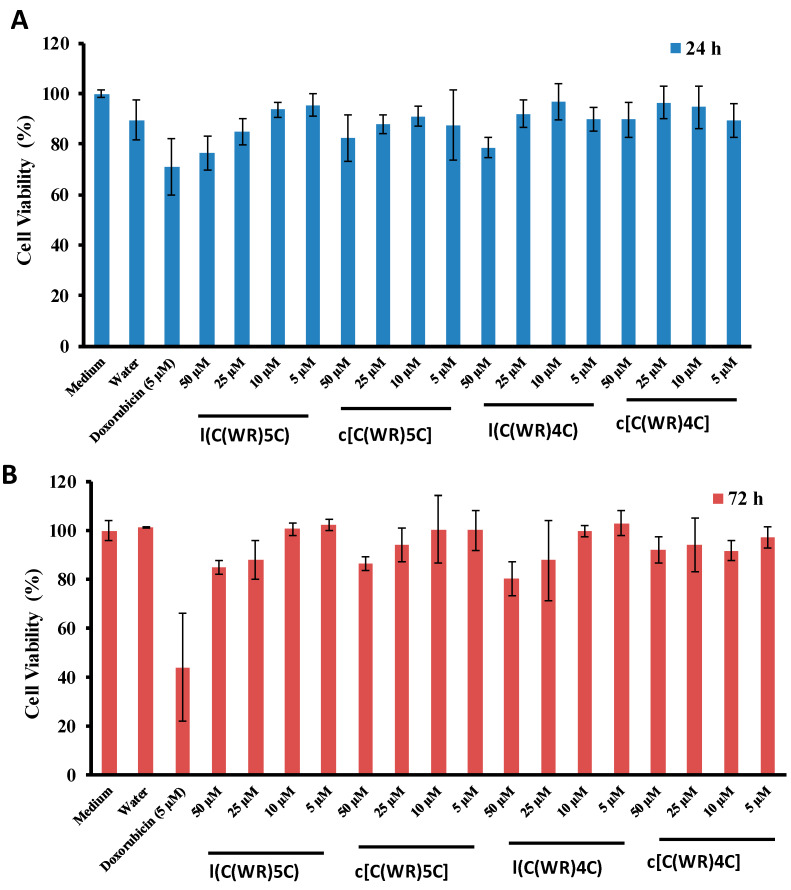
MTS assay of peptides (5–50 µM) on SKOV-3 cell lines: (**A**) 24 h incubation and (**B**) 72 h incubation (*n* = 3). (Results are not statistically significant). Error bars are SD.

**Figure 6:**
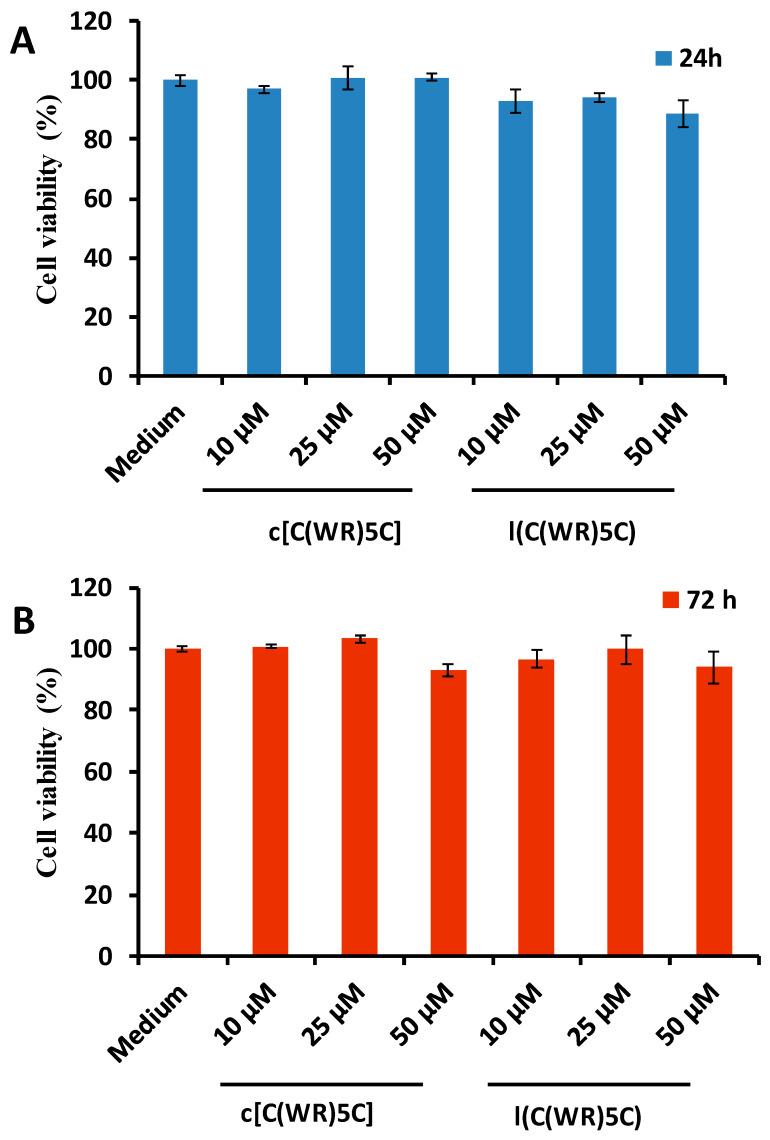
MTS assay of peptides (10–50 µM) on MDA-MB-231 cell lines’ incubation at 24 h and 72 h (*n* = 3) (results are not statistically significant). Error bars are SD.

To evaluate the cytotoxicity of synthesized linear (C(WR)_x_C) and cyclic [C(WR)_x_C] (x = 4–5) peptides, a cell viability assay was employed using kidney (LLC-PK1), leukemia (CCRF-CEM), gastric adenocarcinoma (CRL-1739), breast cancer cell line MDA-MB-231, and ovarian SKOV-3 cell lines at concentrations of 5, 10, 25, and 50 μM at 24 and 72 h (Figure 2, Figure 3, Figure 4, Figure 5 and Figure 6). Doxorubicin (Dox, 5 µM) was used as a positive control. The percentage of cell survival demonstrated that Dox was cytotoxic even at 5 µM. l(C(WR)_5_C) reduced the cell proliferation by 40% and 49%, respectively, after 24 and 72 h incubation in LLC-PK1 cells (at 50 μM), while cell proliferation was reduced with c[C(WR)_5_C] by 53% and 56%, respectively, after 24 h and 72 h incubation (at 50 μM) (Figure 2). Both l(C(WR)_5_C) and c[C(WR)_5_C] showed significant toxicity at 50 µM in CCRF-CEM cells (reduced the cell proliferation by 38% and 51% after 24 h and 27% and 38% after 72 h incubation, respectively) (Figure 3). There was a 48% reduction in cell proliferation in CRL-1739 after 24 h incubation of c[C(WR)_4_C], while the other compounds did not show any significant toxicity at 5–50 μM (Figure 4). All the peptides did not show any significant toxicity in SK-OV-3 cells (Figure 5) and MDA-MB-231 (Figure 6) at the tested concentrations. Based on these data, a nontoxic concentration of synthesized peptides at 25 µM was selected to evaluate the cellular uptake studies.

### 2.3. Cellular Uptake Studies

#### 2.3.1. Cellular Uptake of Fluorescent-Labeled Compounds in the Presence of Synthesized Peptides

**Figure 7:**
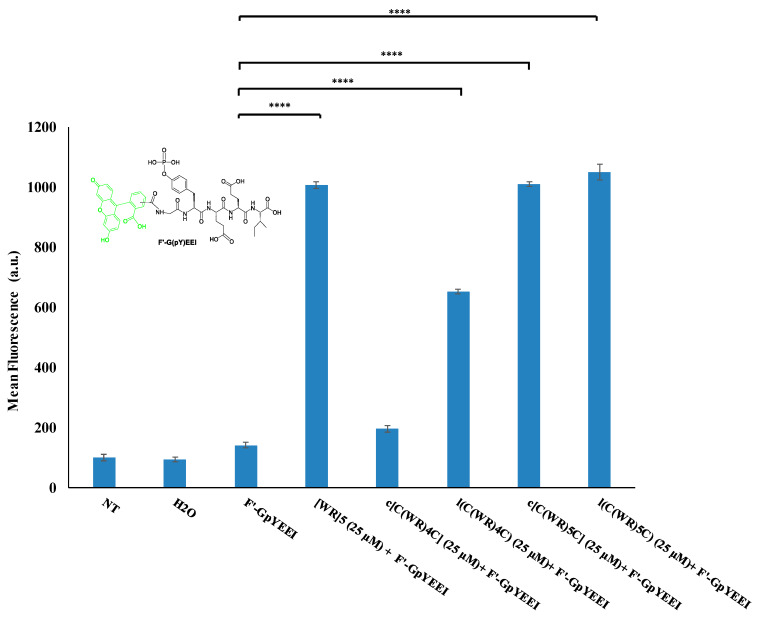
Fluorescence-activated cell sorter (FACS) analysis of F′-GpYEEI (5 μM) using l(C(WR)_5_C) and c[C(WR)_5_C] at 25 µM in CCRF-CEM cells after 3 h incubation (*n* = 3) (if *p* < 0.05 then *, if *p* < 0.01 then **, if *p* < 0.001 then ***, if *p* < 0.0001 then ****). Error bars are SD.

Phosphopeptides are important signaling ligands that are used for the understanding of the activation of different proteins in the cellular processes [31]. However, the phosphopeptides are negatively-charged molecules and are unable to cross the membrane. The SH2 domain ligand of Src tyrosine kinase receptor GpYEEI was found to be an optimal ligand to understand the activation of Src kinase. Therefore, we previously used a fluorescent-labeling version of this phosphopeptide (F′-GpYEEI) for the cellular uptake studies [25]. A nontoxic concentration of synthesized peptides at 25 µM was selected to evaluate the cellular uptake of F′-GpYEEI (5 µM) (F’ = 5(6)-carboxyfluorescein) in CCRF-CEM and MDA-MB-231 cell lines after incubation for 3 h using a fluorescence-activated cell sorter (FACS) analysis. The positive and negative controls were cyclic peptide [WR]_5_ at 25 µM and F′-GpYEEI, respectively (Figure 7). A 7-fold enhancement of the cellular uptake of F′-GpYEEI was detected for both l(C(WR)_5_C) and c[C(WR)_5_C], which were comparable to our previous peptide [WR]_5_ in the CCRF-CEM cell line. c[C(WR)_4_C]) did not show any significant improvement in the cellular uptake of F′-GpYEEI in comparison with [WR]_5_, l(C(WR)_5_C), or c[C(WR)_5_C], while l(C(WR)_4_C) improved the uptake approximately by 4.6-fold.

**Figure 8:**
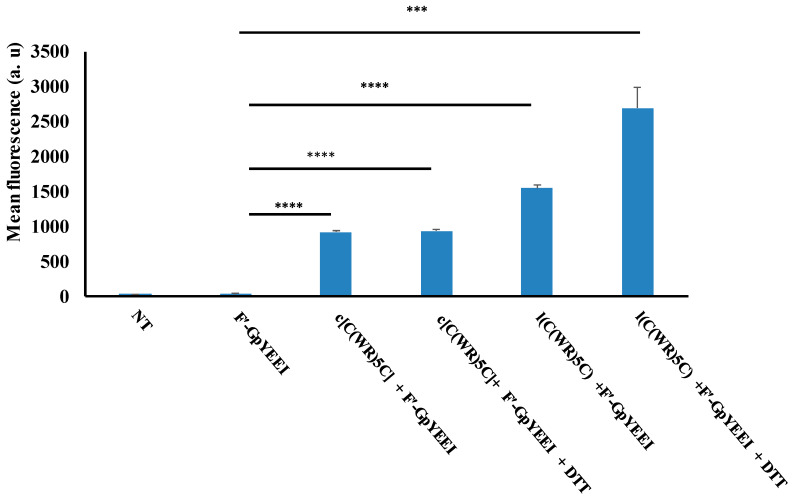
FACS analysis of the uptake of F′-GpYEEI (5 μM) using synthesized peptides l(C(WR)_5_C) and c[C(WR)_5_C] at 25 µM in MDA-MB-231 cells after 3 h incubation in the presence and absence of DTT. NT stands for “No treatment”, *n* = 3, if *p* < 0.05 then *, if *p* < 0.01 then **, if *p* < 0.001 then ***, if *p* < 0.0001 then ****. Error bars are SD.

Peptides l(C(WR)_5_C) and c[C(WR)_5_C] also improved the cellular uptake of F′-GpYEEI (5 μM) significantly in MDA-MB-231 cells. 1,4-Dithiothreitol (DTT) (2 mM) was used to act as the reducing agent of the disulfide bond. Here, the presence of DTT with c[C(WR)_5_C] did not improve the uptake of F′-GpYEEI. On the other hand, the cellular uptake was significantly enhanced in the presence of l(C(WR)_5_C) and DTT (Figure 8), suggesting that the uptake was not dependent on the reduction of the disulfide bridge. The role of DTT in improving the uptake of the linear peptide could be due to the formation of the disulfide bond with the free thiol (SH) groups, generating a different molecular transporter.

**Figure 9:**
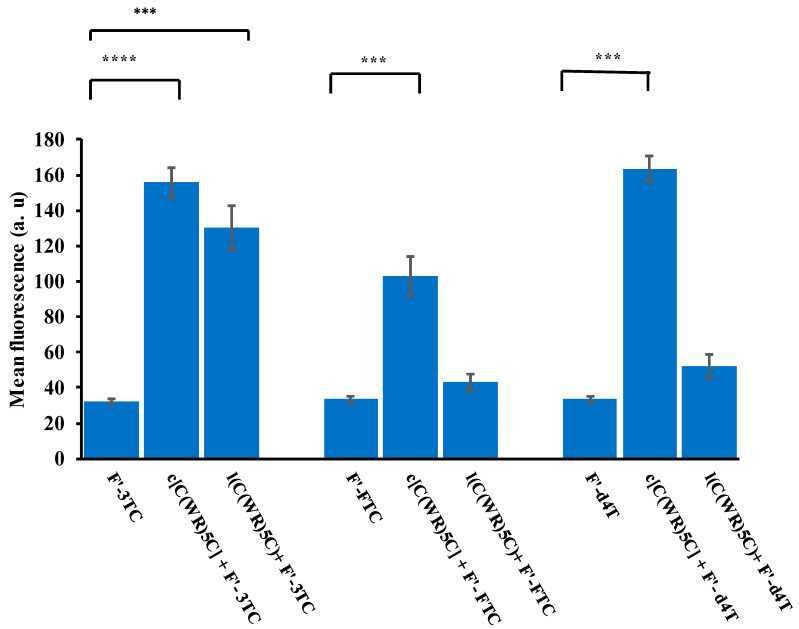
FACS analysis of the uptake of F′-d4T, F′-3TC, F′-FTC, and F’-d4T (5 μM) using synthesized peptides l(C(WR)_5_C) and c[C(WR)_5_C] at 25 μM in MDA-MB-231 cells after 3 h incubation (*n* = 3, if *p* < 0.05 then *, if *p* < 0.01 then **, if *p* < 0.001 then ***, if *p* < 0.0001 then ****). Error bars are SD.

We have previously reported that [WR]_5_ improves the delivery of anti-HIV drugs such as stavudine (d4T), lamivudine (3TC), and emtricitabine (FTC) [17]. The fluorescent-labeled conjugates of these drugs, F′-d4T, F′-3TC, and F′-FTC, were used to determine their cellular uptake in the MDA-MB-231 cell line in the presence of l(C(WR)_5_C) and c[C(WR)_5_C] (Figure 9). The physical mixture of the drug (F′-3TC, 5 μM) with both peptides improved the uptake of the drug in the range of 4.0–4.8-fold. The uptake of F′-d4T (5 μM) was increased when physically mixed with c[C(WR)_5_C]) at 25 μM by 4.9-fold, while l(C(WR)_5_C) was not significantly effective. c[C(WR)_4_C]) at 25 μM enhanced the uptake of F′-FTC (5 μM) by 3-fold, while l(C(WR)_5_C was not effective. Based on these data, the modified peptides were still effective in enhancing the uptake of the anti-HIV drugs, but the improvement was less than that of fluorescent-labeled phosphopeptide in the MDA-MB-231 cell line.

**Figure 10:**
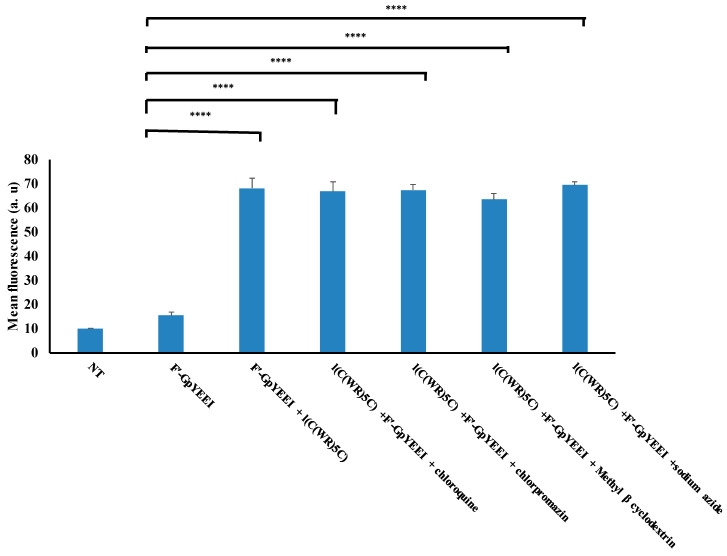
FACS analysis of the uptake of F′-GpYEEI (5 μM) using synthesized peptide l(C(WR)_5_C) (25 µM) in the presence of different endocytic inhibitors in MDA-MB-231 cells after 3 h incubation (*n* = 3, if *p* < 0.05 then *, if *p* < 0.01 then **, if *p* < 0.001 then ***, if *p* < 0.0001 then ****). Error bars are SD.

In order to understand the mechanism of cellular transportation, the cellular uptake of the fluorescent-labeled phosphopeptide was examined in the presence of l(C(WR)_5_C) and different endocytic inhibitors. However, no significant change in the phosphopeptide uptake was observed in the presence of β-cyclodextrin, chlorpromazine, and chloroquine after 3 h incubation, thus eliminating the possibility of clathrin-mediated or caveolae-mediated endocytosis. A comparable uptake was also observed in the presence of sodium azide, suggesting energy-independent endocytosis (Figure 10).

#### 2.3.2. Fluorescence Microscopy

**Figure 11:**
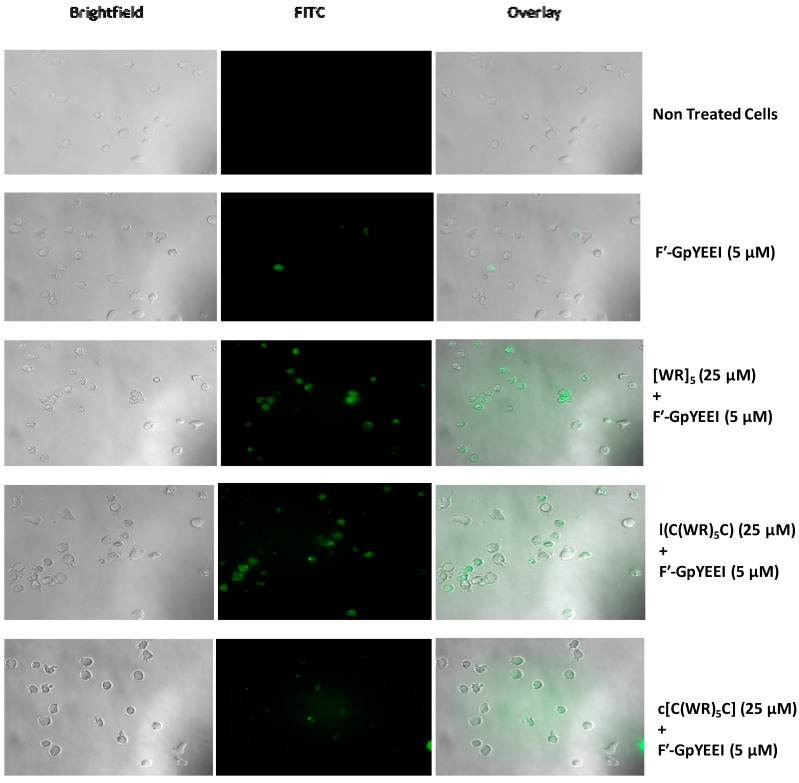
Fluorescence microscope images of peptides l(C(WR)_5_C) and c[C(WR)_5_C]) after 3 h in the CCRF-CEM cells.

The modified and parent peptides l(C(WR)_5_C), c[C(WR)_5_C], and [(WR)_5_] were used at 25 μM to monitor the cellular uptake of F′-GpYEEI (5 μM) using fluorescence microscopy in CCRF-CEM cells. Figure 11 shows the intracellular/cellular localization of the peptides in CCRF-CEM cells after 3 h incubation at 37 °C. l(C(WR)_5_C) demonstrated a significant uptake of F′-GpYEEI, while the uptake was less obvious in the presence of c[C(WR)_5_C]. These data indicate that these modified peptides have different molecular transporter properties in transporting F′-GpYEEI across the cellular membrane. The exact localization of the peptides needs further investigation.

## 3. Conclusions

A series of linear and cyclic peptides l(C(WR)_4_C), l(C(WR)_5_C), c[C(WR)_4_C], and c[C(WR)_5_C] were synthesized after the incorporation of cysteine- and disulfide-assisted cyclization, respectively, to compare the cellular uptake property with the previously synthesized peptide [WR]_5_. The synthesized peptides were found nontoxic at a 5–25 µM concentration in SK-OV-3, CCRF-CEM, and MDA-MB-231 cells. Peptides l(C(WR)_5_C) and c[C(WR)_5_C] improved the cellular uptake of F′-GpYEEI by approximately 7-fold, which was comparable to that of [WR]_5_. The uptake of the fluorescence-labeled phosphopeptide was endocytosis-independent. The delivery of small molecule drugs like d4T, 3TC, and FTC was also improved by 4.0–4.9-fold in the presence of cyclic peptides (c[C(WR)_4_C] and c[C(WR)_5_C]), whereas the linear peptide did not significantly improve the uptake. These data indicate that these peptides have a cell permeation property and could be used in transporting appropriate cargo molecules across the cellular membrane. The delivery of cargo by the cyclic peptide c[C(WR)_5_C] was not dependent on the reduction of the disulfide bridge. This work advances the scientific knowledge in the area of the peptide-based delivery system for improving the cellular uptake of cell-impermeable compounds.

## 4. Materials and Methods

### 4.1. Materials

Cysteine-loaded 2 chlorotrityl resin (H-Cys(Trt)-2Cl-Trt resin) and Fmoc-amino acid building blocks were obtained from AAPPTec (Louisville, KY, USA). Chemical reagents and solvents were purchased from MilliporeSigma (Milwaukee, WI, USA) and used without further purification. Final products were purified using an RP-HPLC system (LC-20AP) from Shimadzu (Canby, OR, USA), with a gradient system of acetonitrile and water with 0.1% TFA (*v*/*v*), and a reversed-phase preparative column (Waters XBridge, BEH130, 10 µm, 110 Å, 21.2 × 250 mm), with a flow rate of 8 mL/min and detection at 214 nm. The analytical RP-HPLC system (LC-20ADXR) from Shimadzu (Canby, OR, USA) was used with a gradient system of acetonitrile and water with 0.1% TFA (*v*/*v*) using a VyDAC column (218TP54, 5 µm, 4.60 × 150 mm), with the flow rate of 1 mL/min and detection at 220 nm. The chemical structures of the final peptides were elucidated using a high-resolution MALDI-TOF (model # GT 0264 from Bruker Inc, Fremont, CA, USA) with α-cyano-4-hydroxycinnamic acid as a matrix in the positive mode. Fluorescence-labeled phosphopeptide (F′-GpYEEI)[25] and fluorescence-labeled anti-HIV drugs [2′,3′-dideoxy-5-fluoro-3′-thiacytidine (FTC) and 2′,3′-didehydro-2′,3′-dideoxythymidine (d4T), and fluorescence-labeled lamivudine (3TC)] were prepared and characterized according to the previously reported procedures [32,33,34,35,36]. [WR]_5_ was prepared according to the previously reported procedure by us [24]. Human leukemia carcinoma cell line (CCRF-CEM, ATCC no. CCL-119), ovarian cancer cell line (SK-OV-3, ATCC no. HTB-77), gastric adenocarcinoma (AGS, ATCC no. CRL-1739), breast carcinoma (MDA-MB-231, ATCC no. HTB-26), and normal kidney cell line (LLC-PK1, ATCC no. CRL-1392) were purchased from American Type Culture Collection (ATCC, Manassas, VA, USA). CellTiter 96^®^ AQueous MTS Reagent Powder was purchased from Promega (Madison, WI, USA). This reagent consists of a tetrazolium compound (3-(4,5-dimethylthiazol-2-yl)-5-(3-carboxymethoxyphenyl)-2-(4-sulfophenyl)-2H-tetrazolium (named MTS) and phenazine ethosulfate (PES) for the cell proliferation studies. All the cell culture materials were purchased from Fischer Scientific (Hanover Park, IL, USA).

### 4.2. Chemistry

#### 4.2.1. Synthesis of Linear Peptides (C(WR)_4_C) and (C(WR)_5_C)

H-Cys(Trt)-2Cl-Trt resin and Fmoc-protected amino acids were used as building blocks for the synthesis of peptides on a scale of 0.3 mmol. 2-(1*H*-benzotriazol-1-yl)-1,1,3,3-tetramethyluronium hexafluorophosphate (HBTU)/*N,N*-diisopropylethylamine (DIPEA) were used as the coupling and activating reagents, respectively. Piperidine in *N,N*-dimethylformamide (DMF) (20%, *v*/*v*) was used for the Fmoc deprotection. H-Cys(Trt)-2Cl-Trt resin (0.3 meq/g, mg) was swelled in DMF under dry nitrogen (15 min × 3 times). The solvent was filtered off. The next Fmoc-protected amino acid (0.9 mmol, 3 equiv.) was coupled to the *N*-terminal of the cysteine in the presence of (HBTU) (0.9 mmol, 3 equiv., 342 mg), and (DIPEA) (1.2 mmol, 6 equiv., 315 µL) in DMF (15 mL) by agitation under dry nitrogen for 1.5 h. The reaction solution was filtered off after the completion of the coupling. The resin was washed with 15 mL DMF (2 × 5 min). Deprotection of Fmoc was performed by using a 20% piperidine/DMF solution (*v*/*v*, 10 mL, 2 × 15 min). The reaction solution was filtered off, and the resin was washed with DMF (15 mL, 2 × 5 min). The subsequent amino acids were coupled and deprotected in a similar manner. Fmoc deprotection was performed on the last amino acid. The resin was washed with DMF (15 mL, 2 × 5 min). The resultant peptides were cleaved from the resin using a cleavage cocktail of TFA/thioanisole/EDT/anisole (90:5:3:2, *v*/*v*/*v*/*v*) for 3 h. The crude products were precipitated by the addition of cold diethyl ether purified by RP-HPLC using a gradient of 0–90% acetonitrile (0.1% TFA) and water (0.1% TFA) over 60 min with a C-18 column. Purified peptides were lyophilized to yield a white powder (100 mg). The chemical structures of the peptides were elucidated using a matrix-assisted laser desorption/ionization (MALDI) time-of-flight (TOF) analyzer.

MALDI-TOF (*m*/*z*) for (C(WR)_4_C) (**1**)**,** [C_74_H_100_N_26_O_11_S_2_]: calcd,1592.7506; found, 1592.7797 [M]^+^.

MALDI-TOF (*m*/*z*) for (C(WR)_5_C) (**2**)**,** [C_91_H_122_N_32_O_13_S_2_]: calcd,1934.9311; found, 1934.2925 [M]^+^.

#### 4.2.2. Synthesis of Cyclic Peptides ([C(WR)_4_C], [C(WR)_5_C])

The linear peptides (**1** or **2**) (30 mg) were dissolved in a 10% DMSO-H_2_O solution (150 mL) using a 250 mL round-bottomed flask. The reaction mixture was stirred in open air for 24 h at room temperature. After the reaction completion as confirmed by the MALDI-TOF mass spectroscopy, the resultant solution was concentrated in a rotatory evaporator to half of its amount. The reaction mixture was further injected directly in RP-HPLC using a gradient of 0–90% acetonitrile (0.1% TFA) and water (0.1% TFA) over 60 min with a C-18 column. Purified peptides were lyophilized to yield a white powder (20 mg).

MALDI-TOF (*m*/*z*) for ([C(WR)_4_C] (**3**), [C_74_H_98_N_26_O_11_S_2_]: calcd, 1590.7350; found, 1590.8999 [M]^+^.

MALDI-TOF (*m*/*z*) for [C(WR)_5_C]) (**4**)*,* [C_91_H_120_N_32_O_13_S_2_]: calcd,1932.9154; found, 1932.9976 [M]^+^.

### 4.3. In Vitro Cytotoxicity Assay of Peptides

The in vitro cytotoxicity of the peptides was evaluated using the human leukemia carcinoma cell line (CCRF-CEM), normal kidney cell line (LLC-PK1), gastric adenocarcinoma (CRL-1739), ovarian (SKOV-3), and breast carcinoma (MDA-MB-231) cell lines to determine the toxicity of the peptides according to the previously reported procedure [17]. CCRF-CEM cells were seeded at 50,000 cells in 0.1 mL per well in 96-well plates. Other cells were seeded at 5,000 cells in 0.1 mL per well in 96-well plates. All cell lines were seeded in RPMI-1640 medium (pH 7.4) containing FBS (10%), 24 h prior to the experiment. The compounds (aqueous solution of peptides) were added to each well in triplicate at a concentration of 5–50 µM and incubated for 24 h and 72 h, respectively, at 37 °C in a humidified atmosphere of 5% CO_2_. Cell culture medium and water were used as negative controls. The peptides were dissolved in water; hence water was used as a negative control to normalize the results. After the incubation period, 20 µL MTS reagent was added to each well followed by incubation of 2 h. The MTS protocol is based on the reduction of MTS tetrazolium by the viable cells. This generates formazan crystals which are soluble in cell culture medium and can be measured at 490 nm. Cell viability was then determined by measuring the fluorescence intensity at 490 nm using a SpectraMaxM2 microplate spectrophotometer (Molecular Devices, San Jose, CA, USA). The percentage of cell survival was calculated as [(OD value of cells treated with the test mixture of compounds)−(OD value of culture medium)]/[(OD value of control cells) − (OD value of culture medium)] × 100%.

### 4.4. Cellular Uptake Studies

CCRF-CEM and MDA-MB-231 cells were used to measure the cellular uptake of F′-GpYEEI, fluorescent-labeled lamivudine (F’-3TC), fluorescent-labeled stavudine (F´-d4T), and fluorescent-labeled emtricitabine (F´-FTC). FACS analysis was performed in order to measure the intracellular uptake of the cargo and determine whether the presence of the peptide affects the uptake of F′-GpYEEI, F´-3TC, F´-d4T, and F´-FTC. The fluorescence intensity was measured in the presence and absence of the synthesized peptides. The cells (5 × 10^8^ cells per well) were taken in 6-well plates in Opti-MEM or serum-free RPMI medium. F′-GpYEEI, F´-3TC, F´-d4T, and F´-FTC at 5 µM were mixed with the synthesized peptides at 25 µM for 30 min and added to the wells for 3 h of incubation at 37 °C. F′-GpYEEI (5 µM), F´-3TC, F´-d4T, and F´-FTC alone (5 µM) were used as negative controls. Additionally, cell culture medium and water were used as negative controls. The peptides were dissolved in water; hence water was used as a negative control to normalize the results. After 3 h, cells were centrifuged at 800 RPM, and the cells were collected as precipitant. Then, the cells were washed twice with PBS. Finally, the cells were resuspended in flow cytometry buffer and analyzed by flow cytometry (FACSCalibur: Becton, Dickinson & Co. (Franklin Lakes, NJ, USA)) using FITC channel and the CellQuest software. The data presented were based on the mean fluorescence signal for 10,000 cells collected. All assays were performed in triplicates. FACS analysis was performed to measure the fluorescence intensity intracellularly and to determine whether the cyclic peptides are facilitating the cargos to cross the membrane.

#### 4.4.1. Mechanism of Cellular Uptake: Effect of Endocytic Inhibitors

Human breast carcinoma cells (MDA-MB-231) were grown in 6-well plates (5 × 10^5^ cells per well) in complete media and then incubated in Opti-MEM. The cells were preincubated with different inhibitors. Chloroquine (100 µM), chlorpromazine (30 µM), or methyl-β-cyclodextrin (2.5 mM) were incubated with cells separately for 30 min followed by incubation with a peptide (25 µM)/phosphopeptide (5 µM) complex for 3 h at 37 °C. To induce ATP depletion, the cells were incubated for 60 min with 0.5% of 75 mM sodium azide in Opti-MEM prior to the addition of the complex. Then, FACS was followed as described above.

#### 4.4.2. Fluorescence Microscopy

CCRF-CEM cells (30 μL of 3 × 10^6^) were seeded with EMEM media overnight on a glass-bottom culture dish. The cells were treated with peptides (25 μM) + F′-GpYEEI (5 μM) in Opti-MEM and incubated for 3 h at 37 °C. After 3 h incubation, cells were centrifuged at 800 rpm for 5 min. The cells were then washed with 400 μL of PBS and centrifuged again. The media was then added to the cells. The cells were then placed on the coverslip and were detected by using a Keyence fluorescence microscope at 40× magnification (BZ-X700. Keyence Corp. of America, Itasca, IL, USA).

### 4.5. Statistical Analyses

To find the statistical significance of the cytotoxicity studies and uptake studies, an ANOVA test was performed using a single factor in MS Excel 2016. For the cytotoxicity assay, cells with the medium were used as control and compared with cells treated with the highest concentration of peptide. In the case of the uptake studies, drug-treated cells were considered as a control and compared with a drug/vehicle combination. A *p* value below 0.05 was considered significant for all analyses and incorporated in the figures.

## Supplementary Materials

The following are available online. The MALDI spectra, analytical HPLC, NMR of selected compounds, and Ellman’s reagent reaction are provided.
