# Supplementary material for: Comparative Molecular Transporter Properties of Cyclic Peptides Containing Tryptophan and Arginine Residues Formed through Disulfide Cyclization
Source: Molecules. 2020 Jun 2;25(11):2581. doi: 10.3390/molecules25112581 (PMC7321319; doi:10.3390/molecules25112581)
Supplement: Supplementary file 1 [file molecules-25-02581-s001.pdf]

Supplementary Materials

Comparative Molecular Transporter Properties of Cyclic Peptides Containing Tryptophan and Arginine Residues Formed through Disulfide Cyclization

Eman H. M. Mohammed,[1,2,#], Dindyal Mandal,[1,#] Saghar Mozaffari,[1] Magdy Abdel-Hamied Zahran,[2] Amany Mostafa Osman.[2,3] Rakesh Kumar Tiwari,[1,*] Keykavous Parang[1,*]

[1] Center for Targeted Drug Delivery, Department of Biomedical and Pharmaceutical Sciences, Chapman University School of Pharmacy, Harry and Diane Rinker Health Science Campus, Irvine, California 92618, USA.
[2] Chemistry Department, Faculty of Science, Chemistry department, Menoufia University, Shebin El-Koam, Egypt 51132
[3] Department of Chemistry, College of Science and Humanities in Al-Kharj, Prince Sattam Bin Abdulaziz University, Al-kharj, 11942, Saudi Arabia.

*Corresponding Authors

Keykavous Parang, PharmD. Ph. D
Chapman University School of Pharmacy
Harry and Diane Rinker Health Science Campus
#262, 9401 Jeronimo Road Irvine, CA 92618, USA
Tel: (714) 516-5489. Fax: (714) 516-5481. E-mail: parang@chapman.edu

Rakesh K. Tiwari, Ph.D.
Chapman University School of Pharmacy
Harry and Diane Rinker Health Science Campus
#263, 9401 Jeronimo Road Irvine, CA 92618, USA
Tel: (714) 516-5483. Fax: (714) 516-5481. E-mail: tiwari@chapman.edu

Figures S1-S4: MALDI-TOF Mass Data of Synthesized Compounds

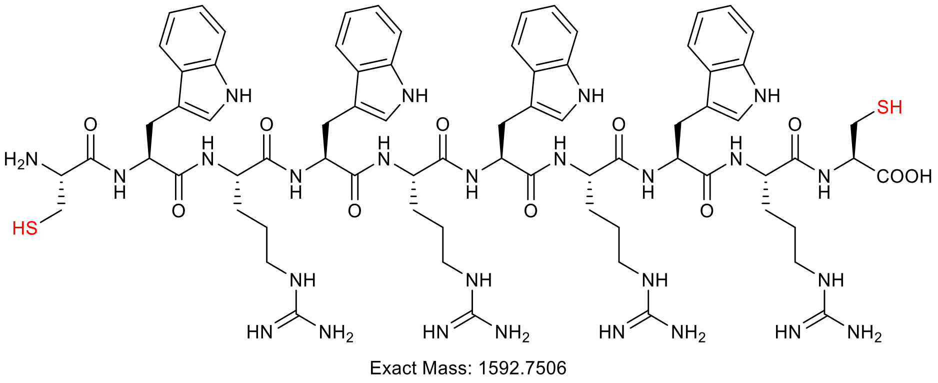

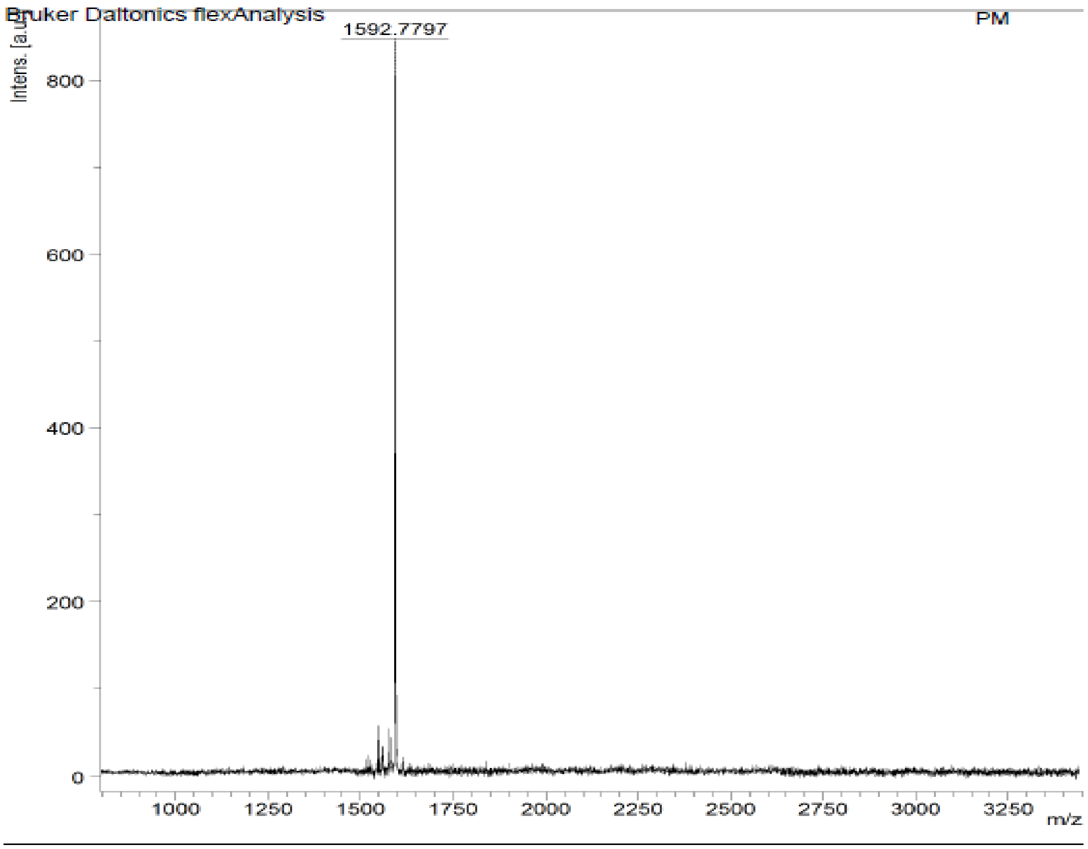

Figure S1. MALDI-TOF (m/z) for ($C(WR)_4C$).

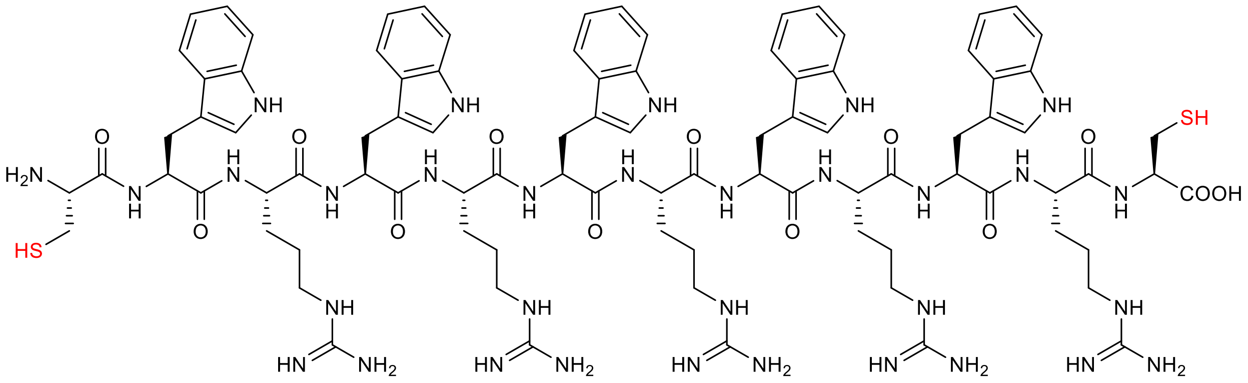

Exact Mass: 1934.9311

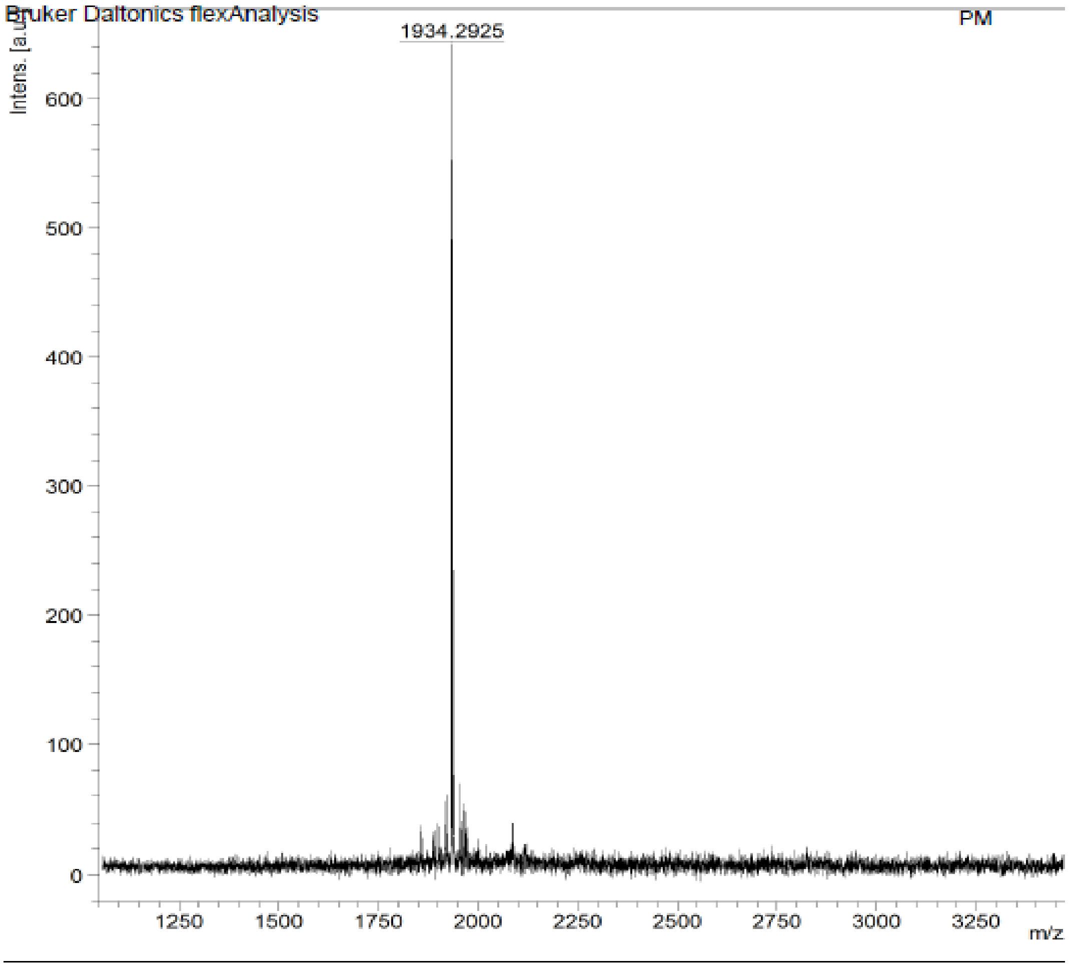

Figure S2. MALDI-TOF (m/z) for (C(WR)$_5$C).

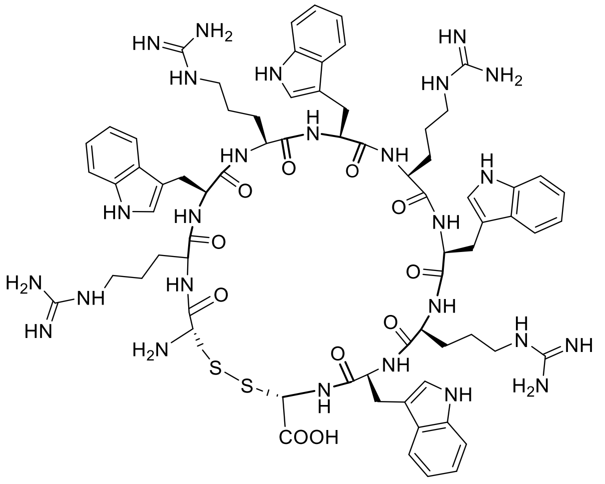

Chemical Formula: $C_{74}H_{98}N_{26}O_{11}S_2$
Exact Mass: 1590.7350

[C(WR)$_4$C]

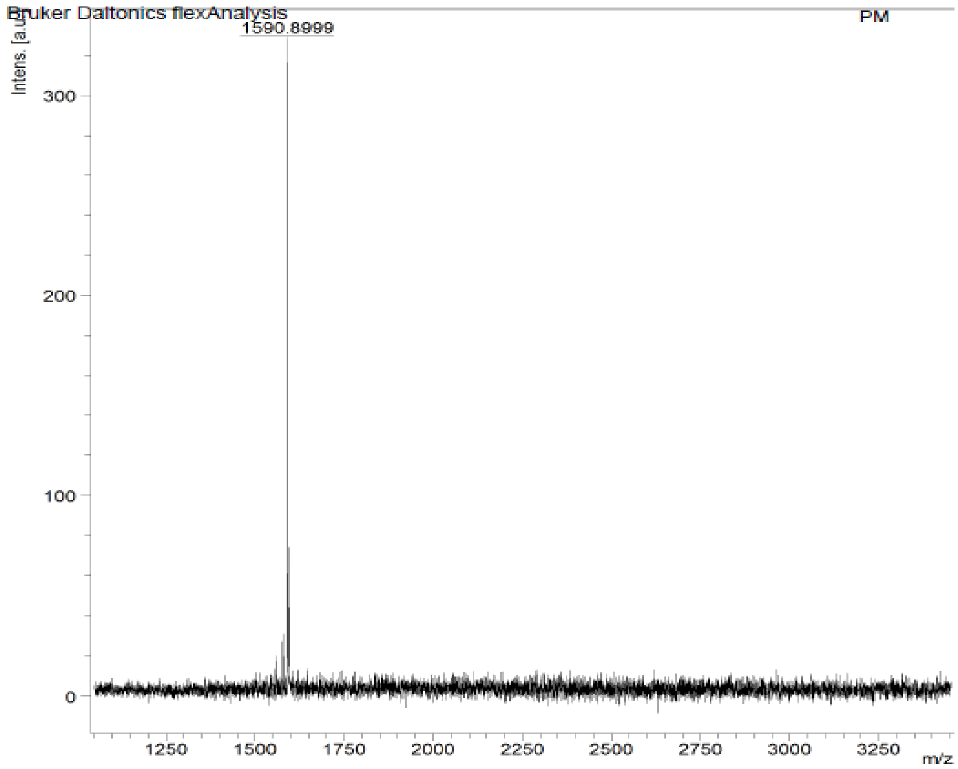

Figure S3. MALDI-TOF (m/z) for [C(WR)$_4$C].

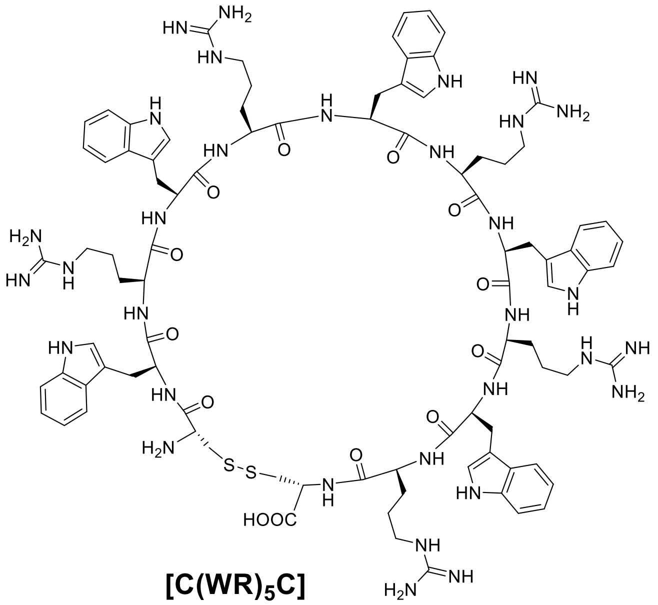

[C(WR)$_5$C]

Chemical Formula: $C_{91}H_{120}N_{32}O_{13}S_2$
Exact Mass: 1932.9154

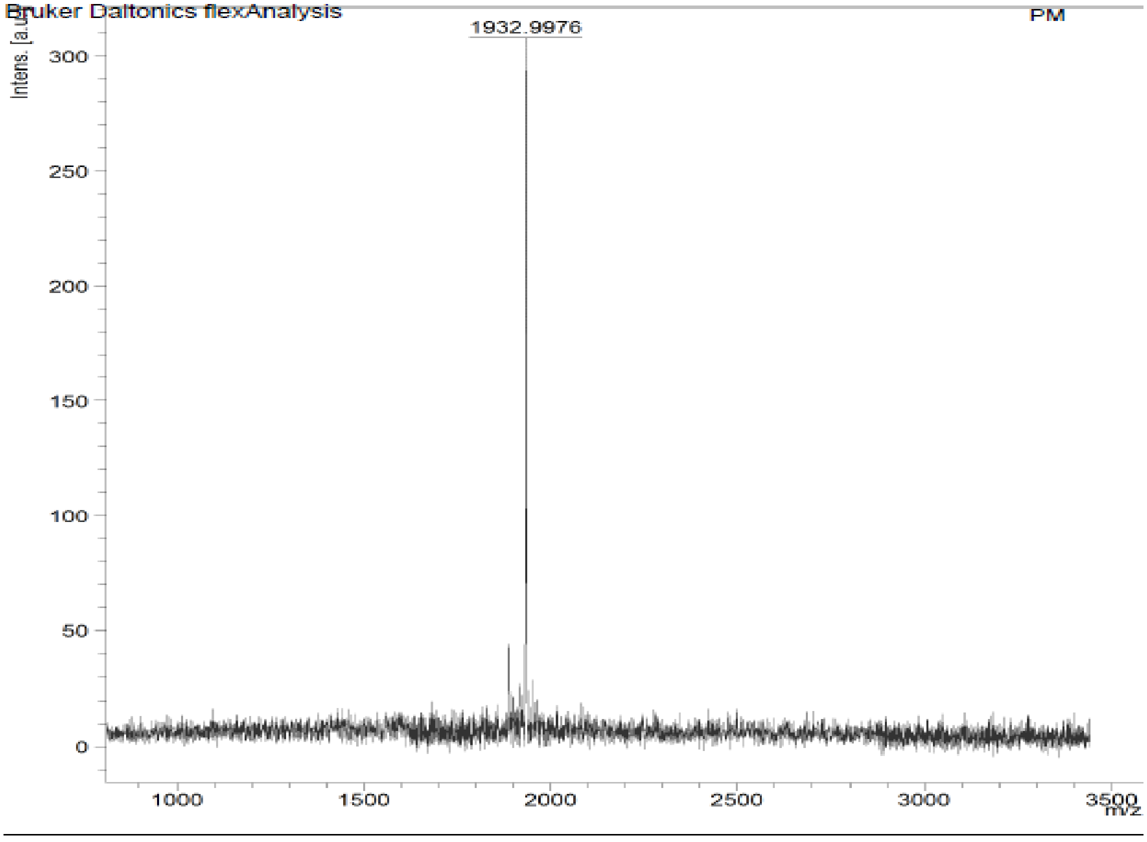

Figure S4. MALDI-TOF (m/z) for [C(WR)$_5$C]

Figures S5-S8: Zoom MALDI-TOF Mass peak of Synthesized peptides (for more proof of disulfide bridge formation).

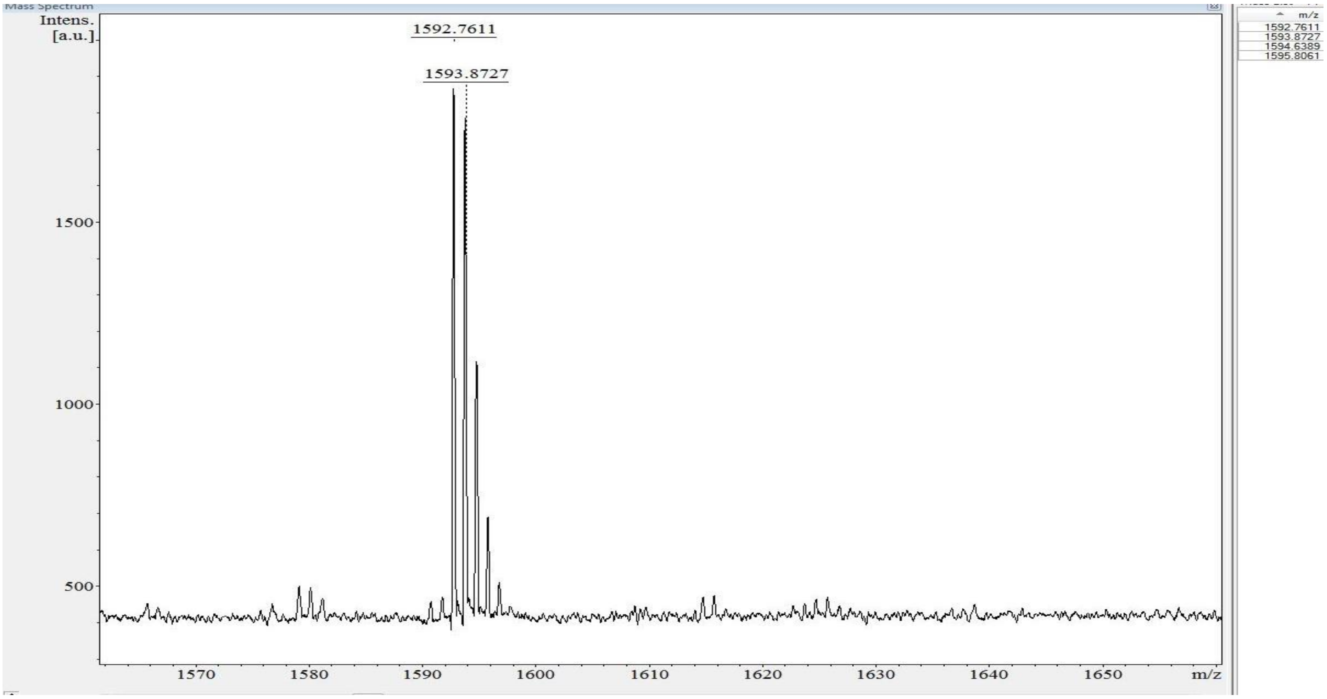

Figure S5. Zoom MALDI-TOF (m/z) for (C(WR)$_4$C).

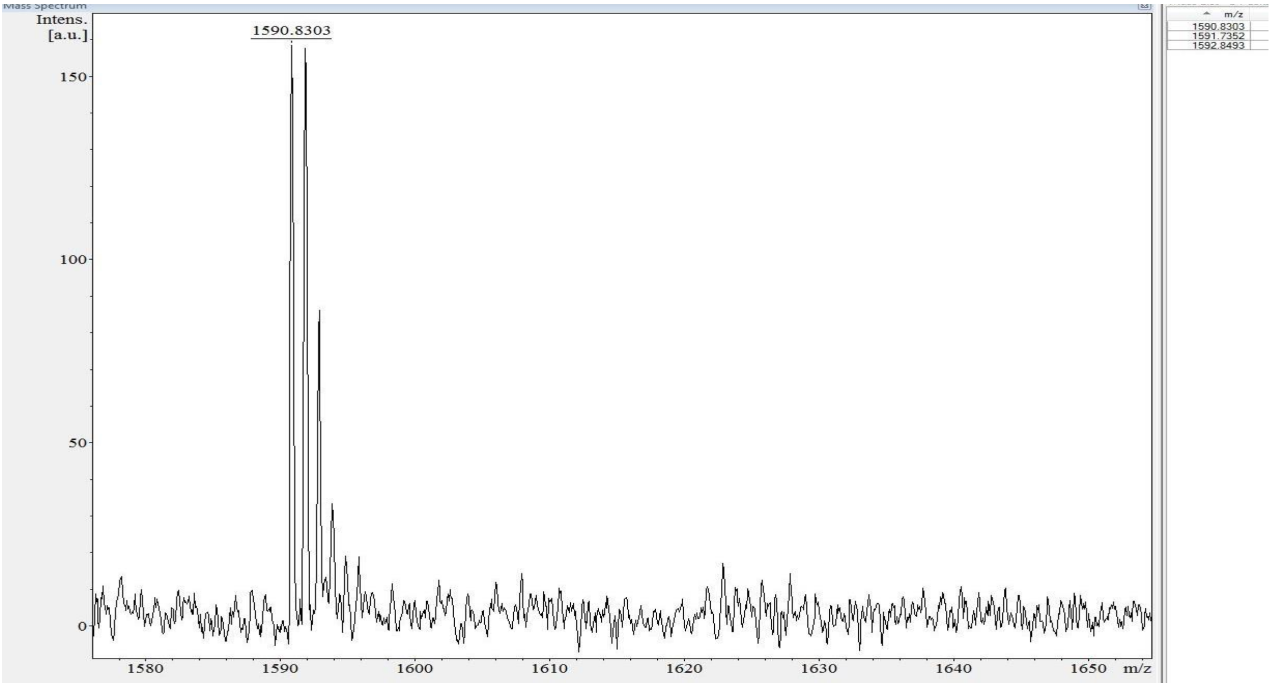

Figure S6. Zoom MALDI-TOF (m/z) for [C(WR)$_4$C].

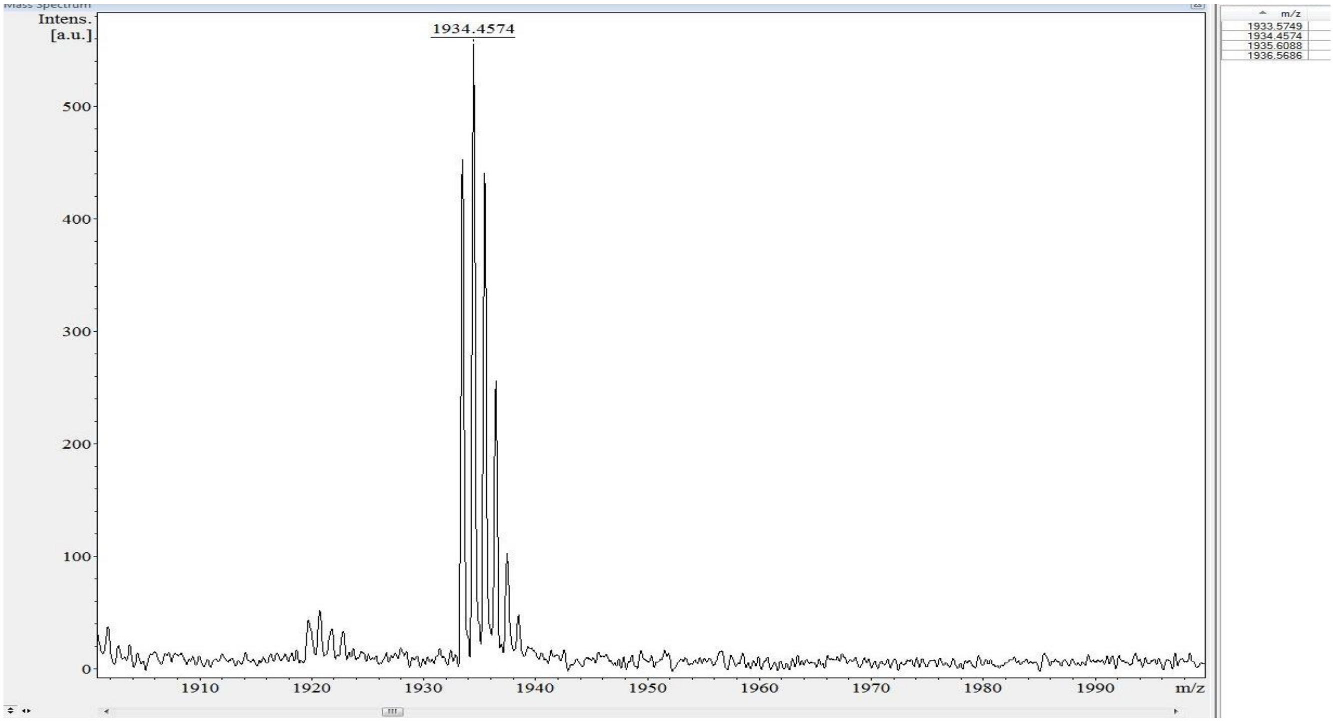

Figure S7. Zoom MALDI-TOF (m/z) for (C(WR)$_5$C).

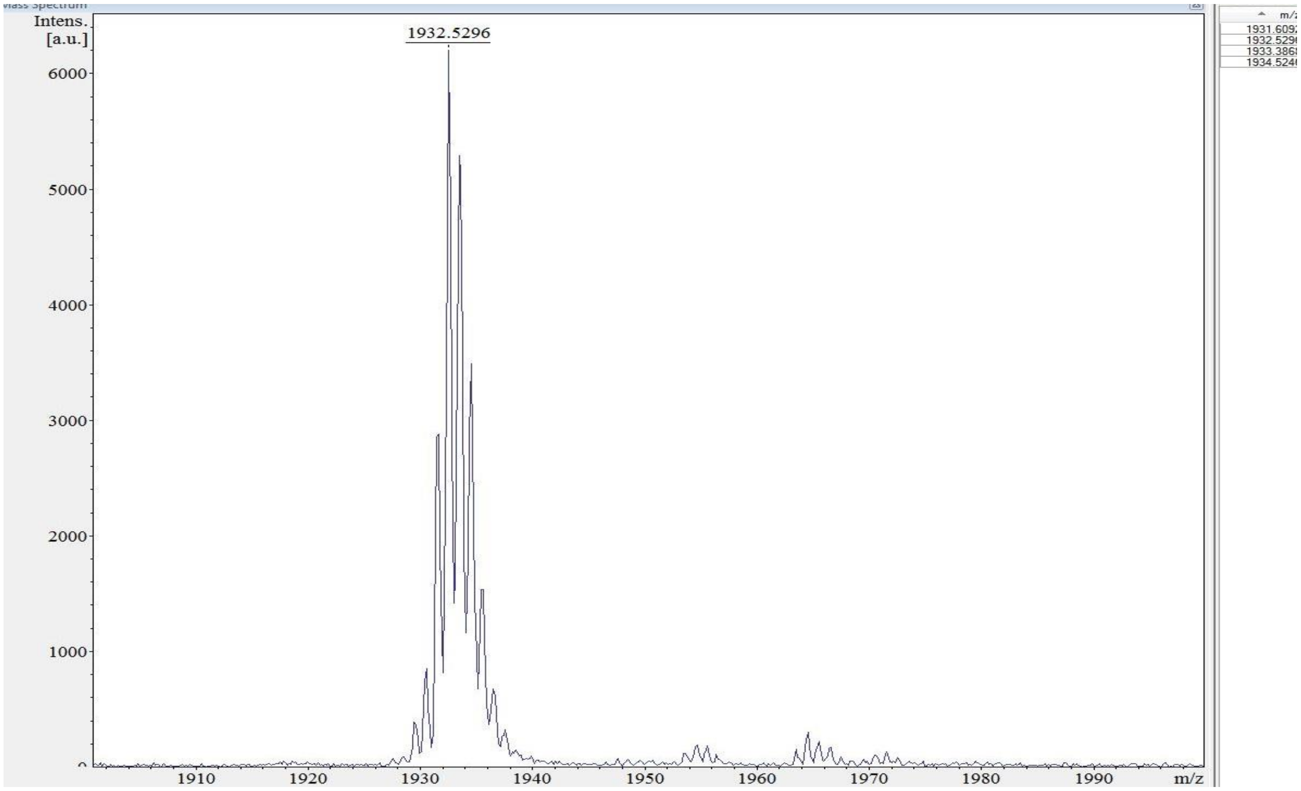

Figure S8. Zoom MALDI-TOF (m/z) for [C(WR)5C]

Figures S9-S12. Analytical HPLC data of linear peptides (C(WR)$_4$C and C(WR)$_5$C) and cyclic peptides ([C(WR)$_4$C] and [C(WR)$_5$C]) peptides. The analytical RP-HPLC system from Shimadzu (LC-20ADXR) with a gradient system of acetonitrile and water with 0.1% TFA (v/v) was used using a reversed-phase VyDAC column (218TP54, 5 µm, 4.60 mm x 150 mm) and flow rate of 1 mL/min with the detection at 220 nm. Analytical HPLC data showed a difference in the retention time (RT). The value between linear and cyclic peptides confirmed the formation of disulfide bridge.

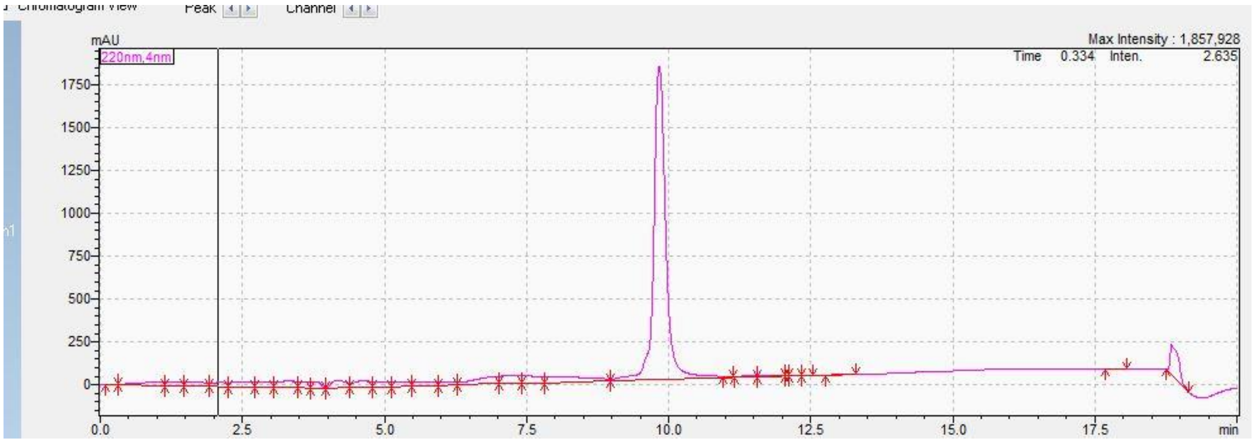

Figure S9. Analytical HPLC chromatogram for linear (C(WR)$_4$C).

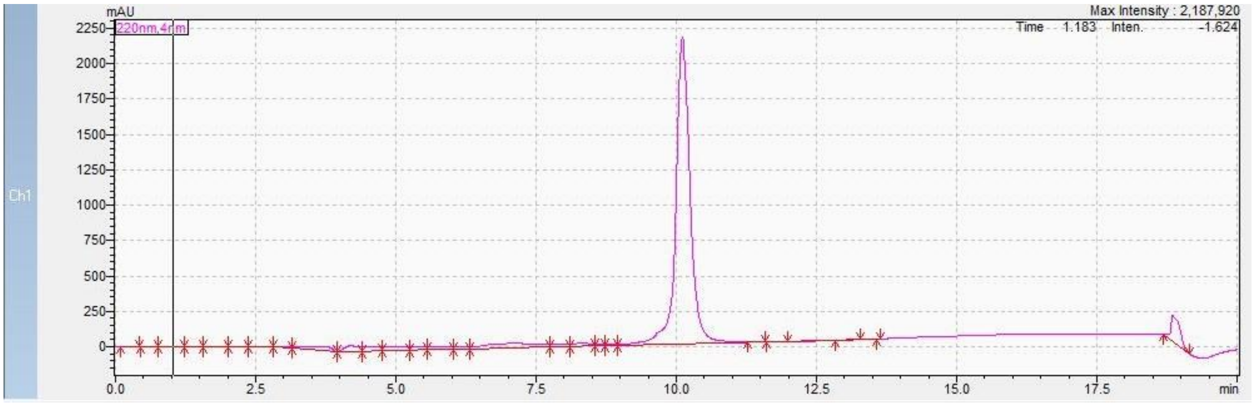

Figure S10. Analytical HPLC chromatogram for cyclic [C(WR)$_4$C].

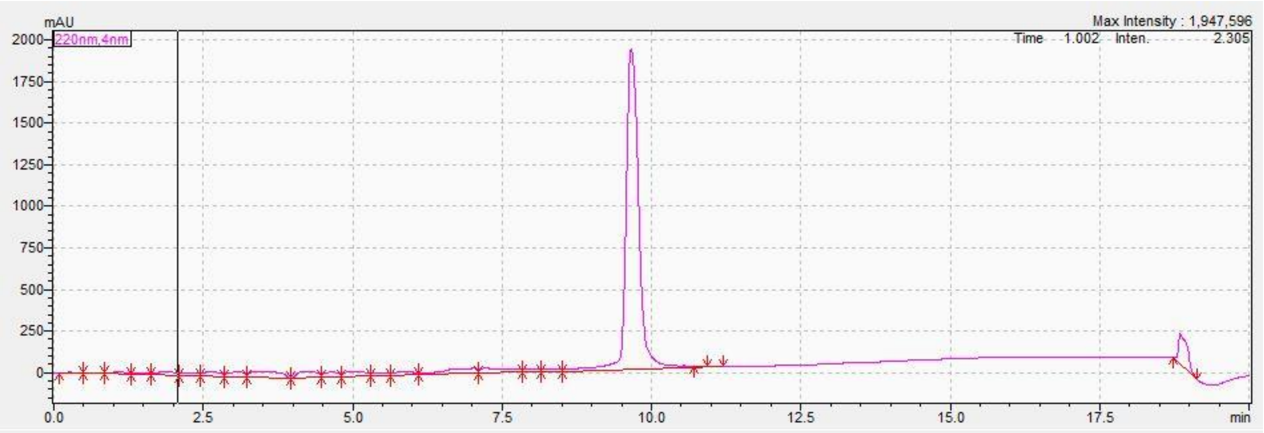

Figure S11. Analytical HPLC chromatogram for linear (C(WR)$_5$C).

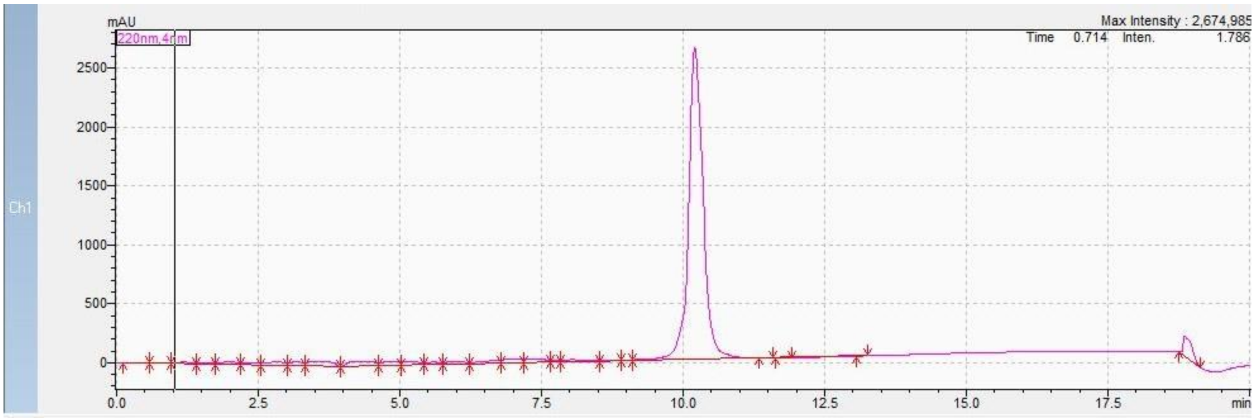

Figure S12. Analytical HPLC chromatogram for cyclic [C(WR)$_5$C].

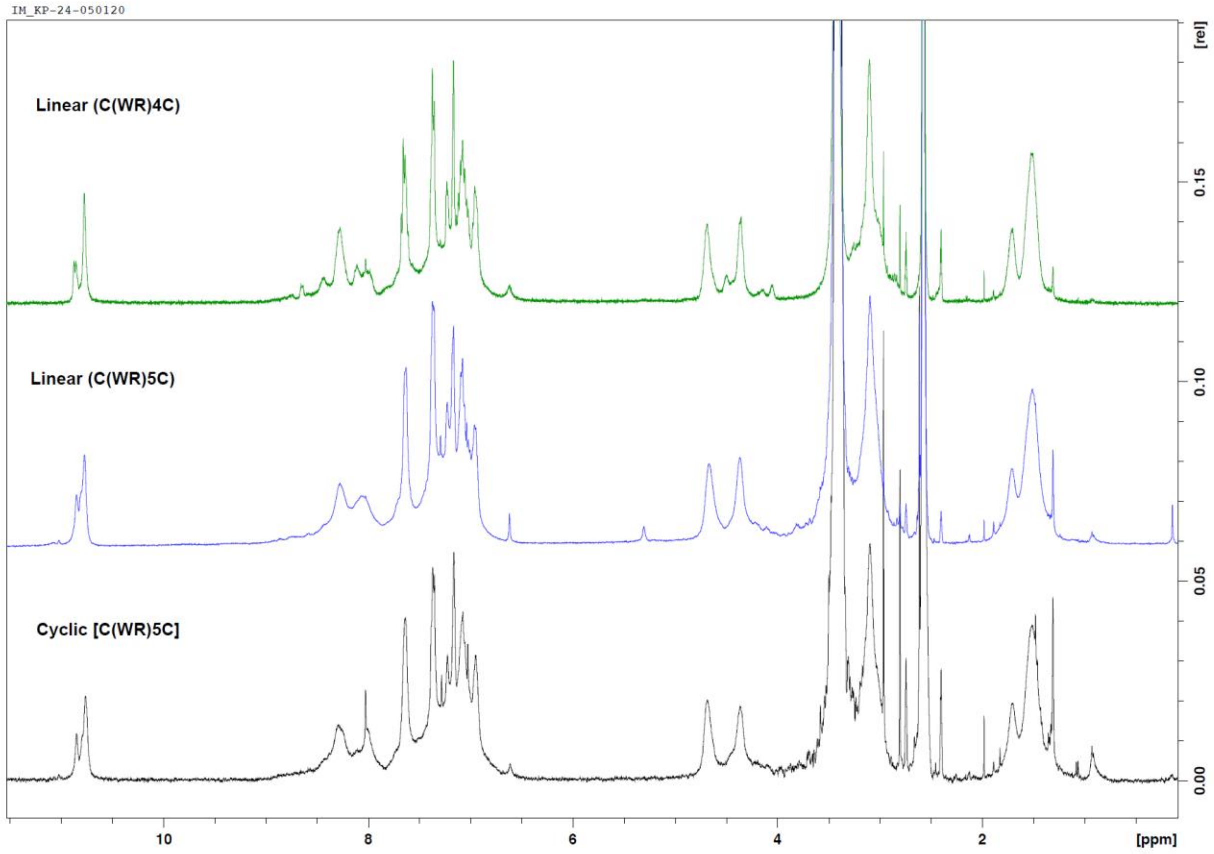

Figure S13. ^{1}H NMR (400 MHz, DMSO-d6) of [C(WR)$_4$C], (C(WR)$_5$C), and [C(WR)$_5$C].

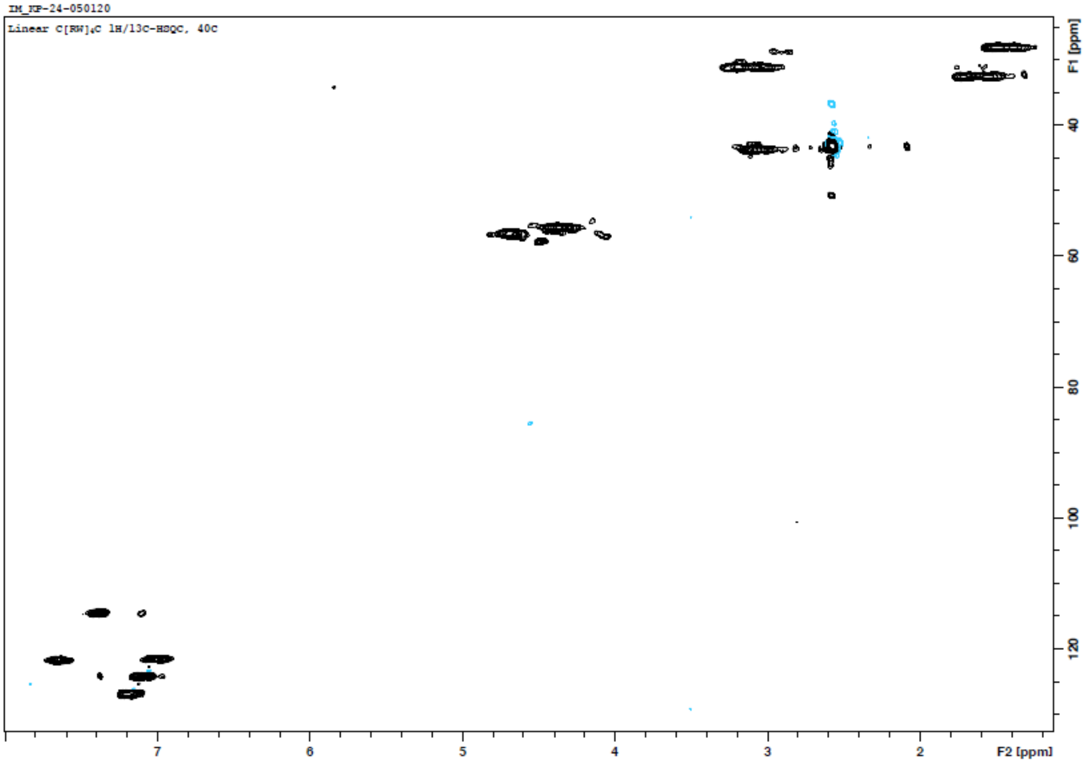

Figure S14. HSQC NMR 1H/13C (DMSO-d6) of linear (C(WR)$_4$C).

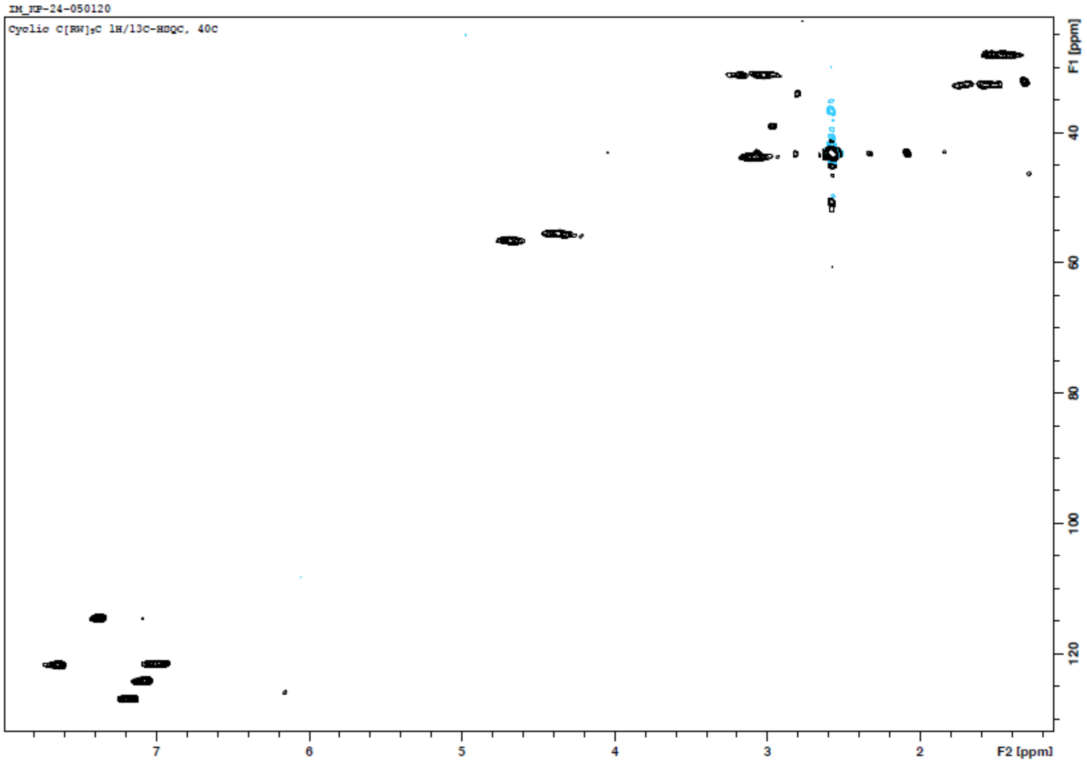

Figure S15. HSQC NMR 1H/13C (DMSO-d6) of cyclic [C(WR)$_5$C].

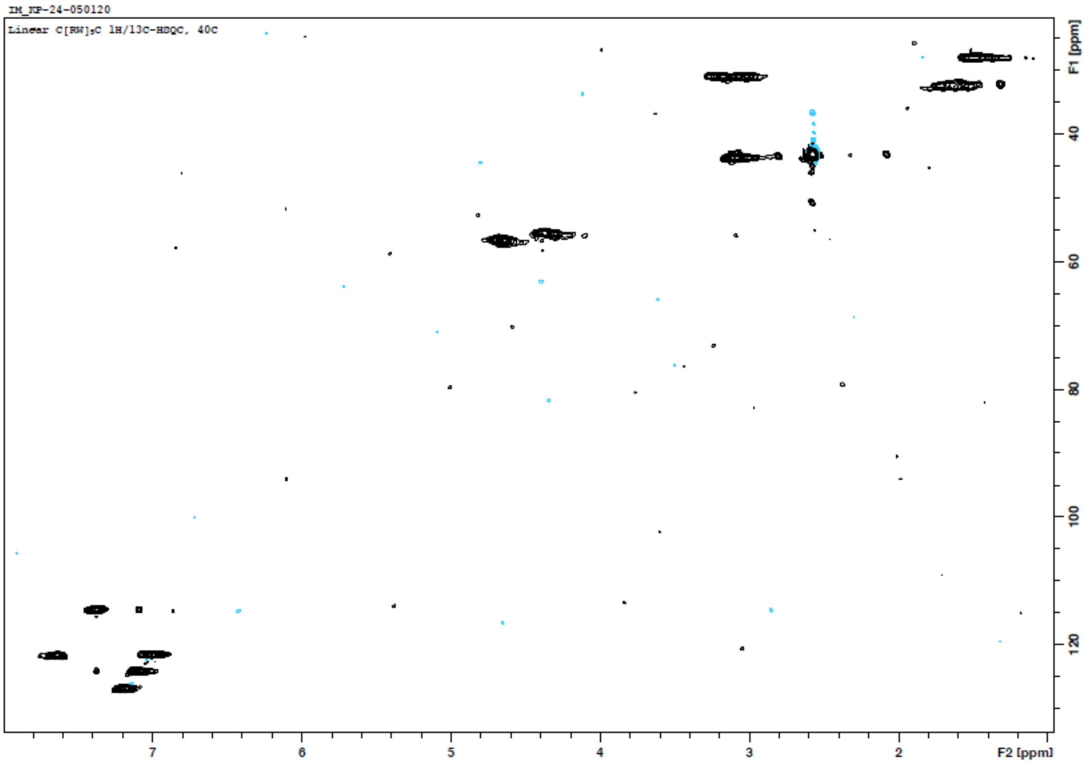

Figure S16. HSQC NMR $^1H/^{13}C$ (DMSO-d6) of linear $(C(WR)_5C)$.

Ellman's Reagent Reaction. Ellman's reagent ((5,5'-dithiobis-(2-nitrobenzoic acid) or DTNB) (4 mg) was dissolved in 1 mL of DMSO. About 150 µL of this solution was added to the peptide solution (1-2 mg in 50 µL water) and the results were monitored visually to confirm the presence of the disulfide bridge in [C(WR)$_5$C]. The cyclic peptides containing the disulfide bridge showed clearly no color change, while a yellow color was observed for the linear peptides containing thiol groups. These data indicated that the cleavage of the disulfide bond in DNTB in the presence of the linear peptides containing thiol group (Figure S17).

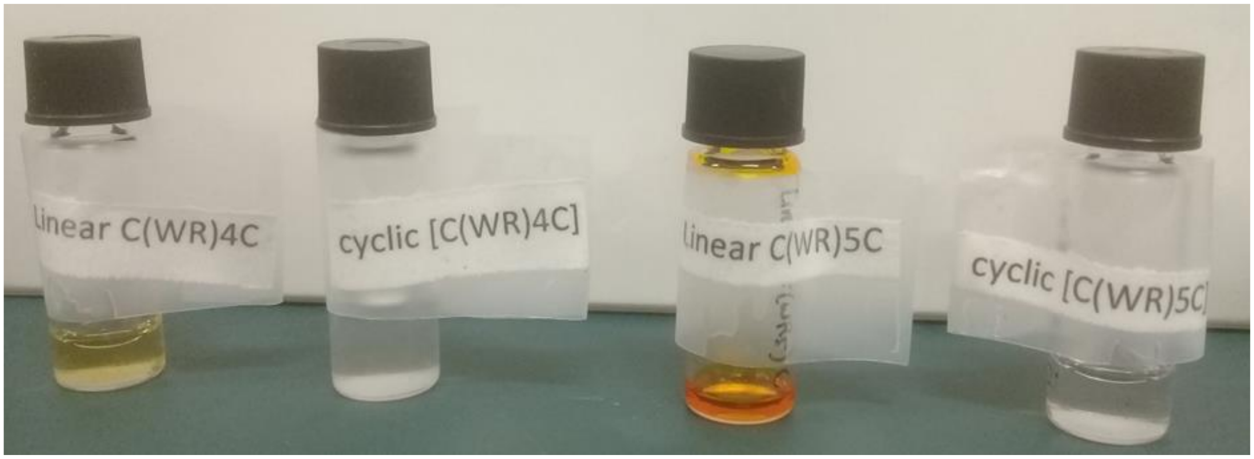

Figure S17. Ellman's reaget reaction with cyclic [C(WR)$_5$C], cyclic [C(WR)$_4$C], linear (C(WR)$_5$C), and linear (C(WR)$_4$C).
